# Randomized Controlled Trial of Visualization versus Neuromonitoring of the External Branch of the Superior Laryngeal Nerve during Thyroidectomy

**DOI:** 10.1007/s00268-012-1547-7

**Published:** 2012-03-09

**Authors:** Marcin Barczyński, Aleksander Konturek, Małgorzata Stopa, Agnieszka Honowska, Wojciech Nowak

**Affiliations:** 1 Department of Endocrine Surgery, 3rd Chair of General Surgery, Jagiellonian University College of Medicine, 37 Prądnicka Street, 31-202 Kraków, Poland; 2 Unit of Otolaryngology, Narutowicz Municipal Hospital, 37 Prądnicka Street, 31-202 Kraków, Poland

## Abstract

**Background:**

Injury to the external branch of the superior laryngeal nerve (EBSLN) during thyroidectomy results in a lowered fundamental frequency of the voice and deteriorated voice performance in producing high-frequency sounds. It remains unclear if the use of intraoperative nerve monitoring (IONM) can improve the clinical outcome of thyroidectomy in terms of preserved individual voice performance. This study was designed to test that hypothesis.

**Methods:**

A total of 210 consenting female patients planned for total thyroidectomy were randomly assigned to two groups equal in size (n = 105): visual inspection of the EBSLN and RLN vs. this plus additional EBSLN and RLN monitoring. The primary outcome was the identification rate of the EBSLN. The secondary outcomes included: anatomical variability of the EBSLN according to the Cernea classification and changes in postoperative voice performance. Voice assessment included pre- and postoperative videostrobolaryngoscopy and an analysis of maximum phonation time (MPT), voice level (VL), fundamental frequency (Fo), and voice quality rating on the GRBAS scale.

**Results:**

The following differences were found for operations without vs. with IONM: identification rate of the EBSLN was 34.3 % vs. 83.8 % (*p* < 0.001), whereas a 10 % or higher decrease in phonation parameters was found in 10 % vs. 2 % patients for MPT (*p* = 0.018), 13 % vs. 2 % for VL (*p* = 0.003), and 9 % vs. 1 % for Fo (*p* = 0.03), a change in the GRBAS scale > 4 points in 7 % vs. 1 % (*p* = 0.03), and temporary RLN injury was found in 2 % vs. 1 % (*p* = 0.56), respectively.

**Conclusions:**

The use of IONM significantly improved the identification rate of the EBSLN during thyroidectomy, as well as reduced the risk of early phonation changes after thyroidectomy.

## Introduction

Phonation changes after thyroidectomy have been reported in many investigations [1–7]. They are considered to be multifactorial in origin and can be a consequence of laryngeal nerve injury or other events during thyroidectomy, including arytenoids trauma after endotracheal intubation, cricothyroid dysfunction, strap muscle malfunction or lesion of the perithyroidal neural plexus, laryngotracheal fixation with impairment of vertical movement, and psychological reaction to the operation [1–7]. Injury to the external branch of the superior laryngeal nerve (EBSLN) can occur during the dissection and clamping of the superior thyroid vessels, and the prevalence of this complication has been reported to range from 0.5 % to 58 % [8, 9]. This injury causes complete paralysis of the cricothyroid muscle, which results in lowered fundamental frequency of the voice and deteriorated voice performance in producing high-frequency sounds [10, 11]. Intraoperative nerve monitoring (IONM) has gained widespread acceptance as an adjunct to the gold standard of visual nerve identification, and this technique can be used to identify both the recurrent laryngeal nerve (RLN) and the EBSLN [12, 13]. However, it remains unclear whether there is any IONM-added value to the clinical outcome of thyroidectomy in terms of preserved individual voice performance. This study was designed to test that hypothesis.

## Materials and methods

### Study design and patient selection

A total of 517 patients were referred to our institution for first-time thyroid surgery between September 2009 and June 2010. Of this group, 217 patients were assessed eligible for recruitment to the study. Seven patients refused their consent, whereas 210 patients signed the informed consent and were finally included. Demographic characteristics, indications for surgery, and types of surgical procedure performed are presented in Table 1. The inclusion criterion consisted of thyroid pathology qualified for first-time bilateral neck surgery in a female patient with small- to moderate-sized goiter (< 100 ml in volume). The exclusion criteria included: male gender, previous neck surgery, unilateral thyroid pathology eligible for unilateral lobectomy, goiter volume > 100 ml, preoperatively diagnosed RLN palsy, abnormal preoperative voice assessment on the GRBAS scale, pregnancy or lactation, age younger than 18 years, high-risk patients ASA 4 grade (American Society of Anesthesiology), and inability to comply with the scheduled follow-up protocol. The patients were randomized into two equal-sized groups (n = 105). In group A, the EBSLNs and RLNs were routinely identified by visualization alone, whereas in group B, the standard practice of attempting to visually identify and preserve the EBSLNs and RLNs was continued supported by adjunct of the IONM system. Four group A versus five group B patients were lost to follow-up. Thus, for the final analysis group A consisted of 101 patients (202 EBSLNs and 202 RLNs at risk), whereas group B consisted of 100 patients (200 EBSLNs and 200 RLNs at risk). All the patients were informed of the intent to use the IONM setup to aid potentially in the identification of the EBSLNs and RLNs and to assess the function of these nerves in half of the operations selected by the study protocol on a random basis. However, the patients were blinded to the relevant group assignment. The primary endpoint of the study was the identification rate of the EBSLN. The secondary endpoints included: anatomical variability of the EBSLN according to Cernea classification, and changes in postoperative voice performance. The voice assessment included pre- and postoperative videostrobolaryngoscopy and analysis of maximum phonation time (MPT), voice level (VL), fundamental frequency (Fo), and voice quality rating on the GRBAS scale. The study was approved by the Institutional Review Board (The Bioethics Committee of the Jagiellonian University).

**Table 1:** Demographic characteristics, indications for surgery, and types of surgical procedures

Variable	EBSLN + RLN visualization	IONM of the EBSLN + RLN	*p* value
Female gender (no., %)	101 (100)	100 (100)	1.0^†^
Mean age (year)	49.7 ± 14.1	50.3 ± 15.3	0.69^‡^
Thyroid volume on ultrasound (mL)	58.4 ± 24.3	59.7 ± 29.1	0.57^‡^
Operative time (min)	82.4 ± 18.2	84.8 ± 16.9	0.39^‡^
Weight of specimens (g)	61.4 ± 34.2	63.5 ± 32.3	0.41^‡^
Disease			
Nontoxic nodular goiter	69 (68.3)	67 (67)	0.84^†^
Thyroid carcinoma	12 (11.9)	13 (13)	0.81^†^
Graves’ disease	5 (4.9)	4 (4)	0.72^†^
Toxic nodular goiter	15 (14.8)	16 (16)	0.86^†^
Procedure			
Total thyroidectomy	101 (100)	100 (100)	1.0^†^
Central compartment clearance	12 (11.9)	13 (13.0)	0.81^†^
EBSLN at risk	202 (100)	200 (100.0)	1.0^†^
RLN at risk	202 (100)	200 (100.0)	1.0^†^

*SD* standard deviation, *EBSLN* external branch of the superior laryngeal nerve, *RLN* recurrent laryngeal nerve, *IONM* intraoperative neuromonitoring

^†^χ^2^ test

^‡^ *t* test

### Randomization

Randomization was performed by computer and sequencing was based on permuted blocks of 2 and 3 to balance the number of patients in the treatment groups. The patients were randomly allocated to one of the treatment groups in a 1:1 ratio. Information on the IONM use remained in consecutively numbered and sealed envelopes, which were stored in a specific box in the operating room. An envelope containing the allocation was added to the patient’s file once he had entered the operating room. In this way, the patient was blinded to the relevant group assignment. Then, the envelope was opened and the surgeon performed the assigned intervention.

### Anesthesia

Operations in both groups were performed under general anesthesia. Two anesthesiologists involved in the study followed a strict protocol, including premedication with IV midazolam and anesthesia induction with fentanyl, thiopental, and suxamethonium at the body mass-dependent dose. After the endotracheal intubation, all the patients were put on mechanical ventilation (sevoflurane and oxygen mixture). No muscle relaxants were used during the operation except for a short-acting one (suxamethonium) for intubation.

### Surgical technique

All the operations were performed by the same experienced endocrine surgeons involved in the study (MB, AK, MS) with a standard Kocher’s skin incision. The strap muscles were dissected in midline but not divided. All the patients in this study underwent total extracapsular thyroidectomy. In each patient, the RLN nerves were exposed, and the branches of the superior and inferior thyroid arteries were divided close to the thyroid capsule (peripheral ligation). In group A, the EBSLNs and RLNs were routinely identified by visualization alone, whereas in group B, the standard practice of attempting to visually identify and preserve the EBSLNs and RLNs continued supported by adjunct of the IONM system. In both groups, the study protocol dictated documentation of the variability of the EBSLN surgical anatomy (according to Cernea classification) [14] and the RLN anatomy, including bifurcations of the nerve.

### IONM technique

The NIM 3.0 system (Medtronic, Jacksonville, FL) was used in all operations of group B patients (105 patients, 210 EBSLNs, and 210 RLNs at risk), with surface electrodes integrated with an endotracheal tube 7.0 in diameter, which was inserted between the vocal folds by an anesthetist under direct vision during intubation. The standardized technique of neuromonitoring of the RLNs was used, including indirect vagal response evaluation at the beginning and at the end of the operation (IONM = L1 + V1 + R1 + R2 + V2 + L2) according to the recommendations formulated recently by the International Intraoperative Monitoring Study Group [12]. The nerves were stimulated using a monopolar electrode and the interrupted stimulation technique at 1 mA, 100 ms impulse duration, and 4 Hz frequency. In case of bifurcated RLN nerves, the assessment included post-stimulation response of each nerve branch based on acoustic evaluation of the signal, EMG response evaluation (latency and amplitude) and the technique of posterior larynx palpation (“laryngeal twitch”). Superior pole dissection and ligation of the superior thyroid artery and veins were performed before gland mobilization. The superior pole vessels were isolated and, if possible, the EBSLN was identified. The vessels were exposed at their penetration point of the thyroid capsule. Neuromonitoring was used to rule out entrapment of the EBSLN during each portion of superior pole dissection by togging the stimulator probe between the tissue and the cricothyroid muscle. The EBSLN was stimulated by applying the stimulation probe directly to the nerve (if seen) or the cricothyroid muscle. A positive signal was determined by observing contractions of the cricothyroid muscle (“cricothyroid twitch”) and in some cases by hearing an auditory signal and an EMG response on the monitor. In group B patients, an attempt was made to indentify the EBSLN in all cases.

### Perioperative management and follow-up

The assessment protocol was strictly followed by the ENT specialist (AH), who was blinded to the patient relevant group assignment. Videostrobolaryngoscopy (VSL) was mandatory in all the patients to access vocal folds mobility, as well as bowing, inferior displacement of vocal fold, regularity and symmetry of the mucosal travelling wave, and degree of glottis closure (before surgery, and on day 1 postoperatively; in case of abnormal findings, reevaluation was done at 3 and 6 months postoperatively). VSL was performed by using a 70° rigid laryngoscope model RLS 9100B (Kay Elemetrics, Lincoln Park, NJ). Vocal cord paresis for 6 months or more following the operation was regarded as permanent palsy. Voice testing was conducted preoperatively, 2–3 weeks, and 3 months after thyroidectomy. Acoustic testing (Kay Elemetrics Computer Speech Lab System, model 4300B with Multi-Dimensional Voice Program software) included: maximum phonation time (MPT, sec), voice level (VL, dB), and fundamental frequency (Fo, Hz). MPT was obtained by having the patient sustain the vowel “a” for as long as possible on a single breath, while observing the display of the computerized oscillogram. The longest of the three attempts was recorded as the maximum phonation time. The degree of dysphonia was evaluated by using the GRBAS scale that documents the perceived grade or overall severity of dysphonia (G), roughness (R), breathiness (B), and asthenia (A), and strained (S) quality of the voice. Each perceptual entity was rated on a 4-point scale, where 0 = normal, 1 = mild, 2 = moderate, and 3 = severe dysphonia [15].

### Statistical analysis

The sample size was estimated based on the principle of detecting a 5 % difference in the incidence of primary or secondary outcome measures with a 90 % probability at *p* < 0.05. The incidence of nerve events was calculated based on the number of nerves at risk. The analysis of both primary and secondary outcomes was performed on an intention-to-treat basis. The statistical significance of categorical variables was evaluated by the χ^2^ test, whereas the Student’s *t* test was used for the evaluation of continuous variables (STATISTICA, Stat-Soft, Katowice). All the data were entered onto a dedicated spreadsheet (Microsoft Excel 2007; Microsoft Corporation, San Jose, CA) by a medical assistant and then analyzed by a statistician. *p* < 0.05 was considered to indicate significance.

## Results

**Fig. 1:**
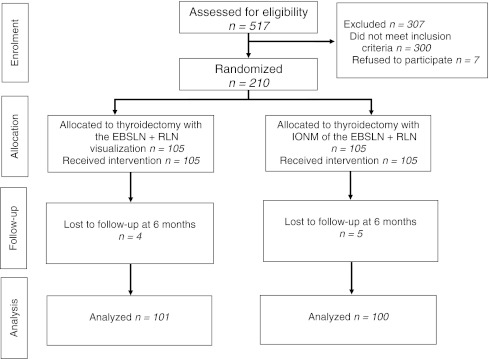
Flow chart of the study. *EBSLN* external branch of the superior laryngeal nerve, *RLN* recurrent laryngeal nerve, *IONM* intraoperative neuromonitoring

A total of 517 patients with goiter qualified for first-time bilateral thyroid surgery at our institution between September 2009 and June 2010 were considered for inclusion into this trial. Three hundred patients were found ineligible, because they met at least one of the exclusion criteria. Seven eligible patients refused their consent. Finally, 210 female patients were recruited and randomized to two groups equal in size: 105 each. All of the recruited patients received the planned intervention. Four vs. five patients were lost to follow-up and 101 vs. 100 patients were finally analyzed (operated on without vs. with IONM, respectively). The flow chart of the study is presented in Fig. 1.

### Primary endpoint analysis

The EBSLN was successfully identified in 72 (34.3 %) of 210 nerves at risk among patients operated on without IONM vs. 176 (83.8 %) of 210 nerves at risk among patients operated on with IONM (*p* < 0.001). Identification of the EBSLN among patients operated on with IONM was confirmed by observed the cricothyroid twitch during stimulation of the nerve in all the patients, but a positive auditory signal and a corresponding EMG waveform was observed in 130 of 176 (73.9 %) identified EBSLN. The mean amplitude of evoked potential during stimulation of the EBSLN was 249.5 ± 144.3 μV, and this value was significantly lower than mean amplitude of evoked potential observed during stimulation of the RLN, which was 638.5 ± 568.4 μV (*p* < 0.001). In three cases of Cernea type 2B EBSLN, the cricothyroid twitch and an EMG response that were present during stimulation of the EBSLN before superior pole thyroid vessels ligation became absent after mobilization of the ipsilateral thyroid lobe. However, in all three cases the nerves were anatomically intact. The RLN was successfully identified in all the patients (100 %) in each group.

### Secondary endpoints analysis

**Fig. 2:**
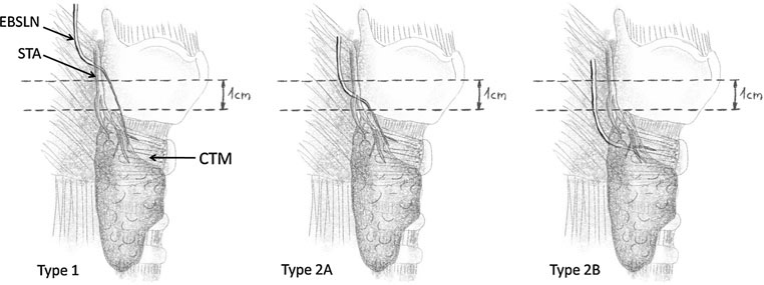
Surgical anatomy of the EBSLN according to Cernea classification. *EBSLN* external branch of the superior laryngeal nerve, *STA* superior thyroid artery, *CTM* cricothyroid muscle

The anatomical variability of the EBSLN found in this study according to Cernea classification (Fig. 2) is presented in Table 2. Identification rate of all types of EBSLN was significantly improved among patients operated with the use of the IONM system, including type 2A and type 2B, which are particularly prone to injury during dissection and ligation of the superior pole thyroid vessels (by 6.7 % and 7.6 %, respectively).

**Table 2:** Incidence of the EBSLN anatomical variations according to Cernea classification and the anatomical variability of the RLN

	EBSLN + RLN visualization	IONM of the EBSLN + RLN	*p* value^†^
EBSLN identification rate (%)	72 (34.3)	176 (83.8)	<0.001
EBSLN not identified	138 (65.7)	34 (16.2)	<0.001
Cernea type 1	26 (12.4)	100 (47.6)	<0.001
Cernea type 2A	20 (9.5)	34 (16.2)	0.04
Cernea type 2B	26 (12.4)	42 (20.0)	0.03
RLN identification rate (%)	210 (100)	210 (100)	1.0
Single-trunk RLN (%)	150 (71.4)	132 (62.9)	0.06
Bifurcated RLN (%)	60 (28.6)	78 (37.1)	0.06
Nonrecurrent laryngeal nerve (%)	0 (0)	1 (0.5)	0.31

Values were calculated for nerves at risk (not for patients)

*EBSLN* external branch of the superior laryngeal nerve, *RLN* recurrent laryngeal nerve, *IONM* intraoperative neuromonitoring

^†^χ^2^ test for all

Prevalence of EBSLN paresis assessed by VSL was significantly higher among patients operated on without vs. with IONM for transient events (5.0 % vs. 1.0 %, *p* = 0.02), but not for permanent events (Table 3). No significant differences were observed in this study for prevalence of RLN paresis, either transient or permanent.

**Table 3:** Incidence of EBSLN and RLN injuries assessed by videostrobolaryngoscopy

	Nerves at risk		*p* value^†^
	EBSLN + RLN visualization (*N* = 202)	IONM of the EBSLN + RLN (*N* = 200)	
EBSLN paresis, *n* (%)			
Transient	10 (5.0)	2 (1.0)	0.02
Permanent	2 (1.0)	1 (0.5)	0.57
Overall	12 (6.0)	3 (1.5)	0.02
RLN paresis, *n* (%)			
Transient	2 (1.0)	1 (0.5)	0.57
Permanent	0 (0)	0 (0)	1.0
Overall	2 (1.0)	1 (0.5)	0.57

Values were calculated for nerves at risk (not for patients)

*EBSLN* external branch of the superior laryngeal nerve, *RLN* recurrent laryngeal nerve, *IONM* intraoperative neuromonitoring

^†^χ^2^ test for all

On functional voice assessment, the following differences were found at 2-3 weeks after surgery for operations without vs. with IONM: a 10 % or higher decrease in phonation parameters was found in 10 % vs. 2 % patients for MPT (*p* = 0.018), 13 % vs. 2 % for VL (*p* = 0.003), and 9 % vs. 1 % for Fo (*p* = 0.03), and a change in the GRBAS scale > 4 points in 7 % vs. 1 % (*p* = 0.03), respectively (details in Table 4). The majority of patients with decreased voice parameters at 2–3 weeks after surgery improved their voice performance at 3 months after operation and the initially observed differences between both study groups became nonsignificant.

**Table 4:** Results of pre- and postoperative functional voice assessment

Variable	EBSLN + RLN visualization (*N* = 101)			IONM of the EBSLN + RLN (*N* = 100)			*p* value^†^
	Preop	2–3 weeks postop	3 months postop	Preop.	2–3 weeks postop	3 months postop	
Decrease >10 % from preop baseline							
MPT	0	10	2	0	2	1	0.018
VL (dB)	0	13	2	0	2	1	0.003
Fo (Hz)	0	9	2	0	2	1	0.03
GRBAS scale (*n*)							
0–3 points	0	24	7	0	13	6	0.08
4–8 points	0	7	2	0	1	1	0.03
9–15 points	0	1	0	0	0	0	0.32

Values represent number of patients

GRBAS scale: overall severity of dysphonia (G), roughness (R), breathiness (B), and asthenia (A), and strained (S) quality of the voice

*EBSLN* external branch of the superior laryngeal nerve, *RLN* recurrent laryngeal nerve, *IONM* intraoperative neuromonitoring, *MPT* maximum phonation time, *VL* voice level, *Fo* fundamental frequency, *preop* preoperative, *postop* postoperative

^†^χ^2^ test for all

## Discussion

IONM has gained widespread acceptance as an adjunct to the “gold standard” of visual nerve identification, and this technique can be used to identify both the RLN and EBSLN. Once the nerve is identified, additional intermittent stimulation of adjacent nonneural tissue versus nerve can help to trace the nerve and all its branches through the dissected field. In addition, electric nerve testing at the end of the operation can serve for postoperative prognostication of nerve function [12]. Despite an increasing number of reports focused on different aspects of IONM in thyroid surgery, it remains unclear whether there is any IONM-added value to the clinical outcome of thyroidectomy in terms of preserved individual voice performance [16–18]. This study was designed to test that hypothesis with special emphasis put on prevalence of temporary EBSLN injury.

The EBSLN carries motor fibers to the cricothyroid muscle, which functions to tilt the thyroid cartilage and tense the vocal cord. Injury to the EBSLN results in changes both in the quality of the voice [19], and the production of high pitched sounds [20]. Clinically, a patient with EBSLN palsy may have a hoarse voice, decreased range of volume, and vocal fatigue and may demonstrate aspiration due to bowing and inferior displacement of the vocal fold [10, 11]. Voice symptoms are more noticeable in women, professional speakers, and, most especially, singers [11].

Several techniques have been described to minimize the potential risk of injury to the EBSLN during superior thyroid vessels dissection and ligation: peripheral ligation of the individual branches of the superior thyroid vessels just on the thyroid capsule, identification of the nerve before ligation of the superior thyroid pole vessels [21], and the use of either a nerve stimulator or intraoperative neuromonitoring [22–26]. Several recently published studies have confirmed that the use of IONM can improve identification rate of the EBSLN, limiting the risk of inadvertent nerve injury [25, 26].

The present study has several advantages. First, a major benefit of IONM of EBSLN documented in this study is assistance in nerve identification, resulting in a significant increase of the EBSLN identification rate and a concomitant decrease in the EBSLN temporary paresis rate. Repetitive stimulations of tissues closely surrounding the nerve can aid the dissection, allowing for nerve localization with a stimulation probe before visual identification (nerve mapping). Visual identification of the EBSLN can be facilitated by the observation of cricothyroid muscle contraction, which is present during direct nerve stimulation. In addition to the observed cricothyroid twitch, in 73.9 % of identified EBSLNs among patients operated with IONM, an auditory signal was present, which was accompanied by an EMG waveform response with the mean amplitude of 249.5 ± 144.3 μV detected by surface electrodes within vocal folds. This response is present due to the existence of the human communicating nerve between the superior laryngeal nerve and RLN, which was described in up to 83 % of anatomical dissection studies [10, 11]. Once the EBSLN nerve is identified intraoperatively, it must be relocated continually during the dissection of the superior thyroid pole vessels to maintain preservation. Some anatomic variants of EBSLN are at an increased risk of injury. In our study, IONM assisted in identifying Cernea type 2A EBSLN in 16.2 % and Cernea type 2B EBSLN in 20 % of the nerves at risk, and these values were significantly higher than 9.5 % and 12.4 % encountered for EBSLN visual identification alone, respectively. This difference can be interpreted as a higher identification rate of EBSLN facilitated by the use IONM. However, some authors argue that the skeletonization and ligation of the upper thyroid vessels close to the thyroid capsule prevents EBSLN injury [21]. A prospective, randomized study by Bellantone et al. did not find any difference in nerve injury rates when comparing the operative technique of skeletonization and individual ligation of the superior pole vessels close to the capsule to the operative technique of EBSLN visual identification before upper pole vessel ligation [21]. However, it is important to keep in mind that Cernea classification type 2B defined by an EBSLN below the plane of the upper border of the superior thyroid pole, which is present in approximately 20 % of cases, exposes the nerve to the highest risk of injury [14, 22]. In a recently published, prospective, randomized trial of nerve monitoring of the EBLN during thyroidectomy under local/regional anesthesia, Lifante et al. found that nerve monitoring aids in the visualization of the EBSLN during mini-incision thyroidectomy and leads to an improvement in patient-assessed voice quality after surgery [25]. Moreover, in the case of giant goiter or big lump within the upper portion of the thyroid lobe, the upper border of the pole is elevated and a probability of encountering a high-risk type 2B nerve increases to 54 % and obtaining a good exposure of this vital nerve structure within the operative field even via a big incision often is troublesome [27].

Another important advantage of this study is that it is a double-blind, randomized, controlled trial, and both patients and ENT specialist who were performing VSL and voice assessment were blinded to the relevant group assignment. This was possible because of the choice of a battery of tests based on acoustic voice analysis (MPT, VL, Fo) and the GRBAS scale, which is based on clinician’s ratings of dysphonia, which is a more reliable and objective tool for voice assessment than other tools based on subjective patient’s ratings of dysphonia (e.g., Voice Handicap Index – 10, etc.) [28]. Our results suggest that temporary injury to the EBSLN may play a role in the early voice changes occurring in patients without RLN palsy. Patients operated on without IONM experienced early voice alterations significantly more frequent at 2-3 weeks after the operation than patients operated with IONM. However, at 3-month follow-up, the differences became nonsignificant, which was consistent with improved VSL findings and acoustic voice analysis data, allowing for finalizing the diagnosis of temporary EBSLN paresis. We strongly believe, similar to Mangano and Dionigi, that further improvement of neuromonitoring technique and development of standardized approach to the EBSLN monitoring is required [29].

The present study also has a few limitations. Visual identification rate of the EBSLN equal to 34.3 % of nerves at risk was relatively low in this study compared with other reports in which this value ranged from 33 % to 93 % [14, 21, 26, 30]. Despite the fact that we tended to completely expose the sternothyroid-laryngeal triangle to facilitate identification of the nerve, we avoided to incise (even partially) the sternothyroid muscle with electrocautery, which was reported to improve visual control of the EBSLN in cases of enlarged thyroid lobe before placing any superior pole sutures [14, 30]. However, division of strap muscles may deteriorate postoperative voice performance and for that reason was avoided in this study to rule out any possible source of bias. Another important issue is that the best way for demonstration of EBSLN palsy is to perform electromyography of the cricothyroid muscle, which was not done in this study. The rationale for this was to avoid an invasive and uncomfortable test, which might compromise patient compliance with follow-up. However, we used VSL instead, which is sufficient from the clinical point of view. Another minor disadvantage of this study is that no patient-based scale for ratings of dysphonia was used and such a scale to a higher extent reflects the patient’s perception of the effects of dysphonia on the quality of life and often indicates the need for intervention. However, to minimize this drawback, speech therapy was offered to all the patients with abnormal findings encountered during the follow-up.

In conclusion, the EBSLN is at a high risk of injury during dissection of the superior thyroid pole in the course of thyroidectomy in approximately one-third of patients with Cernea type 2A and 2B nerves. Nerve stimulation can objectively identify the EBSLN, leading to a visible cricothyroid muscle twitch in all cases. The use of IONM significantly improved the identification rate of the EBSLN during thyroidectomy, as well as reduced the risk of early but not permanent phonation changes following thyroidectomy.
